# Supplementary material for: The global costs of extreme weather that are attributable to climate change
Source: Nat Commun. 2023 Sep 29;14:6103. doi: 10.1038/s41467-023-41888-1 (PMC10541421; doi:10.1038/s41467-023-41888-1)
Supplement: Supplementary file 1 — Supplementary Information [file 41467_2023_41888_MOESM1_ESM.docx]

The Global Costs of Extreme Weather that are Attributable to Climate Change

## Supplementary File on data collection

Rebecca Newman^1^ and Ilan Noy^2^

(August 2023)

# ^1^Reserve Bank of New Zealand ([rebecca.newman@rbnz.govt.nz](mailto:rebecca.newman@rbnz.govt.nz)).

# ^2^Victoria University of Wellington ([ilan.noy@vuw.acn.nz](mailto:ilan.noy@vuw.acn.nz)).

The data collection process formed a substantial portion of this research. Given that there was no existing database of global FAR measurements, and additionally no database with the matching economic cost data, this data had to be collected before any analysis could occur.

### *FAR data*

The FAR measurements for individual extreme weather events were gathered from a review of the extreme event attribution literature. The starting source for accessing a wide range of this literature was the CarbonBrief^59^ Google Sheet. This spreadsheet compiles papers that attribute weather events to climate change, including a mixture of published scientific papers and rapid studies. A copy of this CarbonBrief spreadsheet is available in the supplementary material. The results from these attribution studies, and the details of the events they study, are not recorded in the spreadsheet. Studies from the CarbonBrief sheet were not examined for this research if:

1. The study recorded inconclusive results of anthropogenic climate change; as these do not provide any useful insight into the human-induced cost of extreme weather events.
2. Studies analysing events with no direct link to economic damages or losses. These include studies about sunshine hours, ocean/marine events, coral bleaching, river flow measures, and ecosystem functioning.
3. Studies attributing global events or weather trends; because economic costs are not clearly linked to events with either large spatial or temporal scope.
4. Studies that did not use a FAR metric or a transformable measure such as a risk ratio to ensure a consistent methodology could be applied.

Once the collection of studies was refined, as per these criteria, the remaining papers were read and key data compiled. This was an extensive process which involved reading over 200 climate attribution papers to, firstly, determine if the paper contains a FAR or transferable metric that could be used in this research; and, secondly, extract key information about the event study and how it was defined. The data collected from each study included countries for which the event was relevant, the spatial and temporal definition used to study the event, the nature of the event, and the FAR measurement. If the study did not include a FAR directly, it was calculated from the risk ratio ($FAR=1-{RR}^{-1})$), or from the provided event probabilities for a factual and counterfactual climate. This data is available in the Combined sheet in the economic attribution spreadsheet provided in the supplementary material.

### *Economic data*

Economic cost data was collected for the extreme weather events for which a FAR was found in the attribution literature. A hierarchy of sources was used to gather economic data, as follows: EM-DAT, DesInventar, estimates from academic literature, estimates from national or international governmental organizations, and, finally, estimations from non-governmental organizations or media reports.

Given that EM-DAT was the primary source of economic data for extreme weather events, their categorizations were adopted for wider data collection. EM-DAT data covers four key variables, but only two were used in our analysis, the number of deaths caused by the event, and the amount of economic damage. EM-DAT defines this as the damage caused to livestock, property, and crops. It includes both uninsured and insured economic damages. The EM-DAT definition is thus similar to the aforementioned definition of direct economic loss from the UNDRR Intergovernmental Expert Working Group on Indicators and Terminology relating to disaster risk reduction.

The economic data collected from other sources did not always fit directly into these cost categories. However, since by far most estimates are of direct losses, any monetary estimates from sources outside of EM-DAT were recorded. The economic data in EM-DAT is recorded in US dollars at the time of the event occurring. To allow accurate aggregation, all economic cost data has been adjusted for inflation to reflect the average price of the US Dollar in 2020. Cost estimates provided in alternative currencies were also exchanged to reflect the 2020 USD. Unless otherwise specified, all results will be stated in USD (2020 value).

Additionally, all the data provided by EM-DAT on the economic costs of extreme weather events over 2000-2019 was collected to input into the extrapolation of a global climate change cost estimate. All data covering heatwaves, droughts, precipitation/floods, storms, wildfires, and cold events covering the study period were collected and formatted in a separate database. This data was collected separately irrespective of whether the event had a matched FAR study.
